# Supplementary material for: Design of highly efficient deep-blue organic afterglow through guest sensitization and matrices rigidification
Source: Nat Commun. 2020 Sep 23;11:4802. doi: 10.1038/s41467-020-18572-9 (PMC7511363; doi:10.1038/s41467-020-18572-9)
Supplement: Supplementary file 1 — Supplementary information [file 41467_2020_18572_MOESM1_ESM.pdf]

Supplementary Information

Design of Highly Efficient Deep-blue Organic Afterglow through Guest Sensitization and Matrices Rigidification

Xu *et al.*

Contents

1. Supplementary Methods S3

2. Supplementary Figures S5

3. Supplementary Tables S19

4. Supplementary Notes S23

Supplementary References S24

1. Supplementary Methods

Materials and solvents

Unless other mentioned, all the materials used in the experiments, including cyanuric acid (**CA**), trimesic acid (**TMA**), isophthalic acid (**IPA**), terephthalic acid (**TPA**) and phthalic acid (**PA**), were purchased from Energy chemical. All the organic solvents were obtained from Eyes Chemical without further purification.

Measurements

Steady-state photoluminescent and phosphorescent spectra, fluorescent and phosphorescent lifetimes and time-resolved emission spectra (TRES) were measured on an Edinburgh FLS 980 fluorescence spectrophotometer equipped with a xenon arc lamp (Xe900), a microsecond flash-lamp (μF900) and a picosecond pulsed light emitting diodes with several wavelength. The absolute photoluminscent quantum yields were also measured on FLS 980 equipment with the help of an integral sphere. Only the phosphorescent emission band (from 365 to 620 nm) was involved to calculate PhQY. Excitation-phosphorescence mapping spectra were measured using Hitachi F-4600 under ambient conditions. Raman spectra were measured on a Horiba-HR-800 Raman spectroscopy system equipped with a 532 nm diode laser source. Solution ^{13}C nuclear magnetic resonance (NMR) spectra were measured on a Bruker Avance III 400 MHz instrument using DMSO-d6 (J&K) as solvent. Solid state ^{13}C NMR spectra were collected using a 9.4 T Bruker Avance III spectrometer with a 4.0 mm MAS probe operating at 100 MHz at room temperature. The absorption spectra were recorded by a Jasco V-750 spectrometer. Powder X-ray diffraction (XRD) patterns were measured using a Bruker D8 Advance diffractometer (Cu Kα: λ = 1.5418 Å) under ambient conditions. Differential scanning calorimetry (DSC) analyses were performed on a NETZSCH DSC214 polyma with a heating rate of 10°C·min^{-1} and a nitrogen flow rate of 50 cm^3·min^{-1}. The photographs were taken by a Nikon D7100 camera under ambient conditions. The application of water-jet printed lifetime-encrypted paper was carried on a HP DeskJet 1111 printer.

The measurement of phosphorescent quantum yield (PhQY). The total photoluminescent quantum yield can be measured by an integral sphere, from which PhQY can be figured out as follows. Fluorescence of both **CA** and **TMA** are located before 350 nm which is in line with the references[14,15]. Thus it can be easily distinguished from phosphorescence of **CT5-20** peaked at 406 nm. Besides, the fluorescence of **CT5-20** is very weak and almost indistinguishable compared to the phosphorescence in its steady-state PL spectrum (Fig. 1e) and the fluorescence decay curves (Fig. 2g). Therefore, we can accurately measure the PhQY by only integrating the emission band in the range from 365 to 620 nm which are only the phosphorescent emission.

Theoretical calculation

The density functional theory (DFT) and time-dependent density functional dependent theory (TD-DFT) calculations were performed to investigate the singlet/triplet exciton transformation using Gaussian 09 package. The functional of M06-2x was adopted to optimize the ground state (S_0) geometries of all molecules

in assistant with the cc-pVDZ basis set. The optimized structures were further characterized by harmonic vibrational frequency analysis to confirm that real local minima without any imaginary frequency was reached at the same computational level. The energies of the lowest singlet excited state (S_1) and triplet excited states (T_n) were computed by M06 suite of density functionals (M06-2X/cc-PVDZ) based on the optimized ground state structure. With the cam-B3LYP functional and cc-pVDZ basis set, spin-orbit coupling (SOC) constants between S_1 and T_n were calculated by the quadratic response function methods using the Dalton program based on the lowest triplet excited state (T_1) structure optimized byTD-M06-2X/cc-PVDZ.

Preparation

CA-based organic afterglow composites: The **CA**-based composites including **CT** (**TMA** dispersed in **CA**), **CI** (**IPA** dispersed in **CA**), **CTP** (**TPA** dispersed in **CA**) and **CP** (**PA** dispersed in **CA**) were prepared in a standard procedure. Take **CT5** as a typical example. Firstly, 5 mg **TMA** was dissolved in 5 mL of deionized water to obtain a 1.0 mg/mL aqueous solution of **TMA**. Secondly, to 1 mL **TMA** solution was added 0.20 g **CA** powder and the mixture was ultrasonicated for 10 min at room temperature. Finally, the mixture was dried by removing the solvent of water under vacuum at 40°C for 24 h to give the composite of **CT5-0** in powder with **TMA** content of 5 wt‰. To obtain **CT5-20** with 20 wt% water content, 25 µL water was added to 0.1 g **CT5-0** followed by ultrasonication at room temperature for 10 min. **CA**-based composites with other guest concentrations and water contents were prepared in the same procedure.

The preparation of rewritable lifetime-encrypted paper. 500 mg **CA** and 5 mg **TMA** were dissolved in 5 mL dimethyl sulfoxide (DMSO) to afford the coating solution. This solution was uniformly coated to the filter paper and dried for 2 h at 100°C to obtain a rewritable lifetime-encrypted paper.

Writing process: The desired pattern was printed by a commercially available printer (HP DeskJet 1111) using water as ink.

Erasing process: The pattern printed on the rewritable paper was erased with DMSO vapor generated by heating DMSO to 120°C, which is much lower than its boiling point. The complete erase of the pattern costs about 15 min (Supplementary Figure 16).

The preparation of white organic afterglow composite. Typically, 99 mg **CT5-20** and 1 mg **DPhCzT**[1] crystal were mixed and ground in an agate mortar at room temperature for 10 min to obtain the white organic afterglow composite.

2. Supplementary Figures

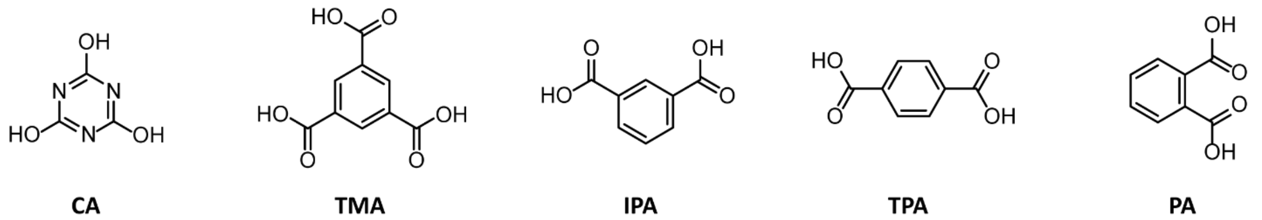

Supplementary Figure 1. Chemical structures of **CA**, **TMA**, **IPA**, **TPA** and **PA**.

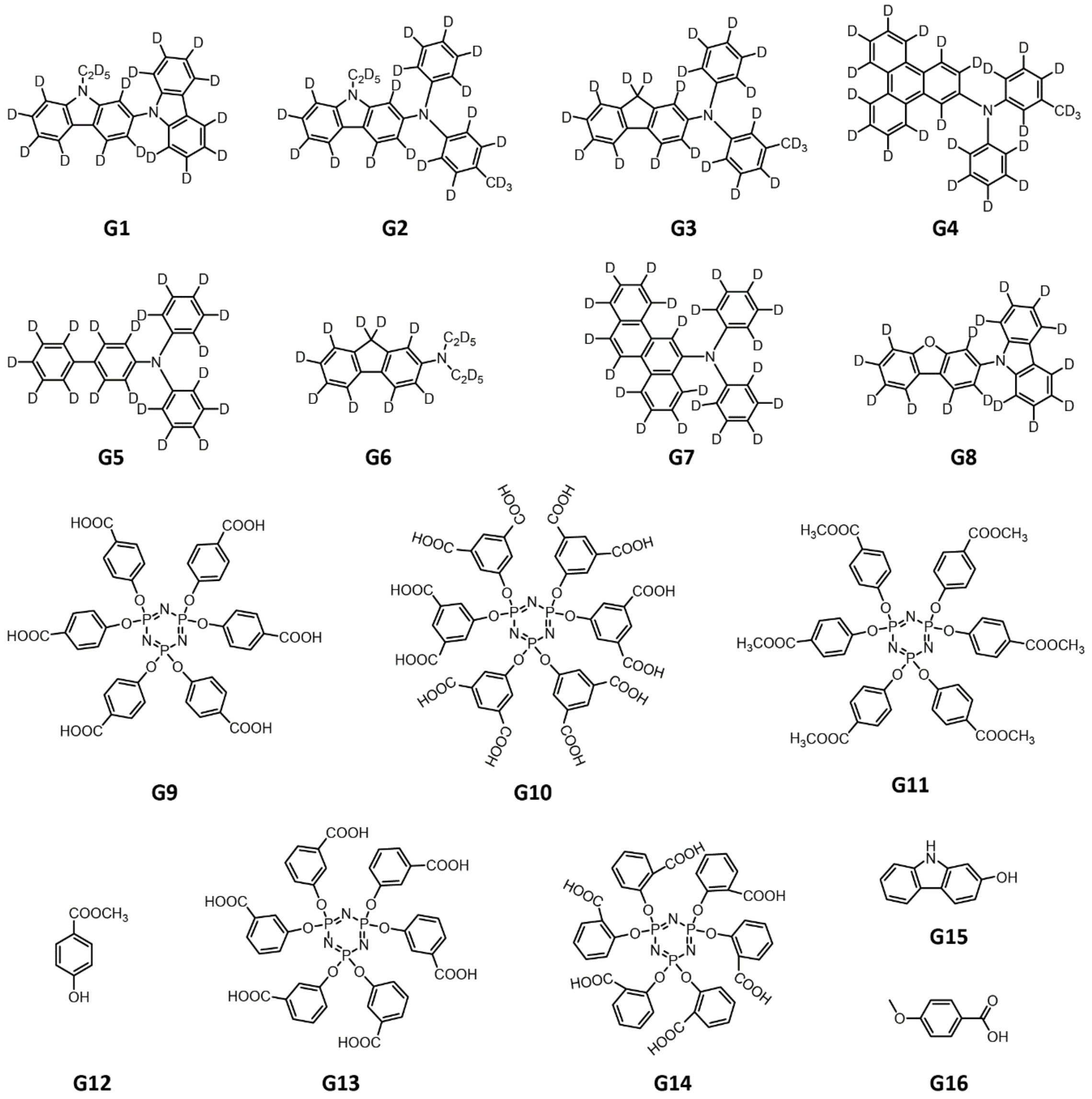

Supplementary Figure 2. Chemical structures of guest materials listed in Supplementary Table 1.

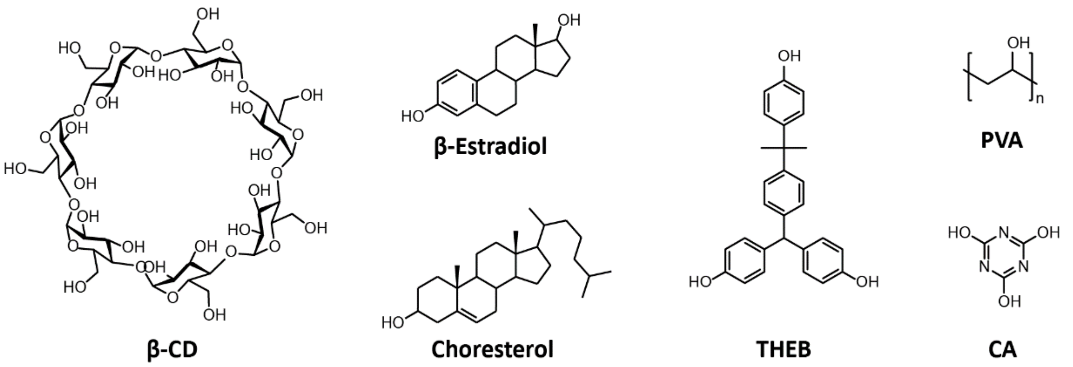

Supplementary Figure 3. Chemical structures of the host materials listed in Supplementary Table 1.

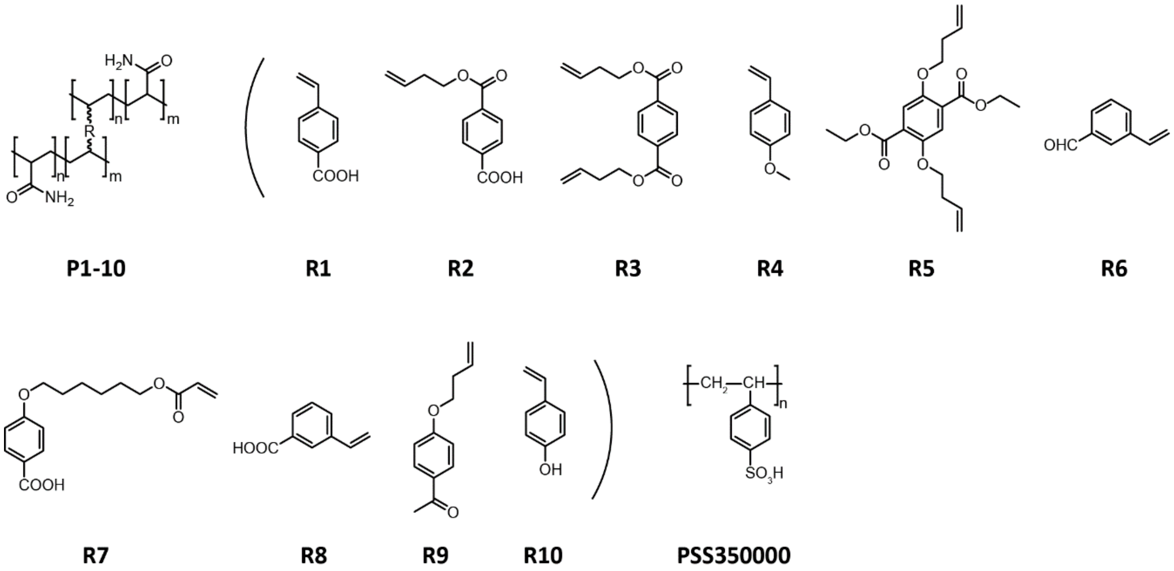

Supplementary Figure 4. Chemical structures of polymer afterglow materials listed in Supplementary Table 1. Structures of monomers are shown in parenthesis.

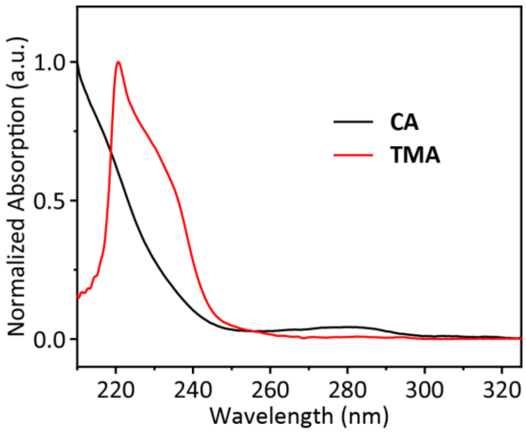

Supplementary Figure 5. Normalized UV-vis absorption spectra of **CA** and **TMA** in ethanol solution (10 μM).

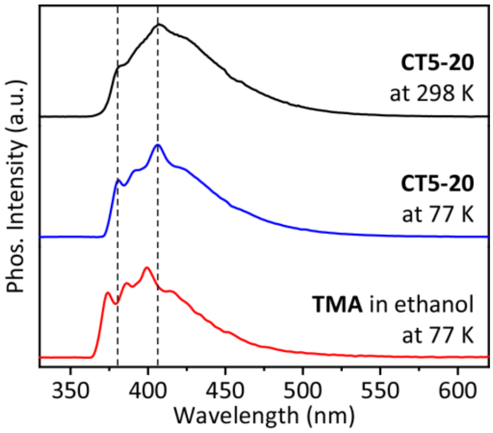

Supplementary Figure 6. Phosphorescent spectra of **CT5-20** powder at 298 K (black) and 77 K (blue) excited by 248 nm, and **TMA** in ethanol at 77 K (red) excited by 288 nm. These spectra were recorded with a delay time of 30 ms.

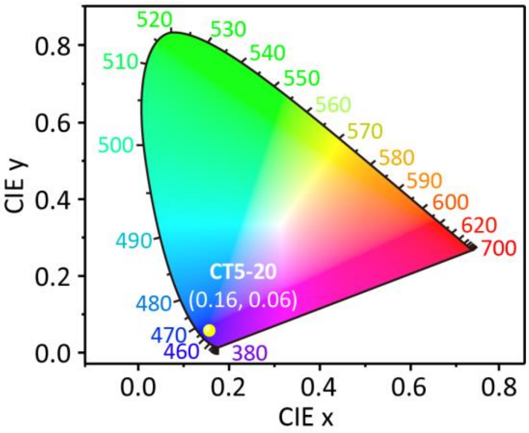

Supplementary Figure 7. CIE coordinate of OURTP emission of **CT5-20**.

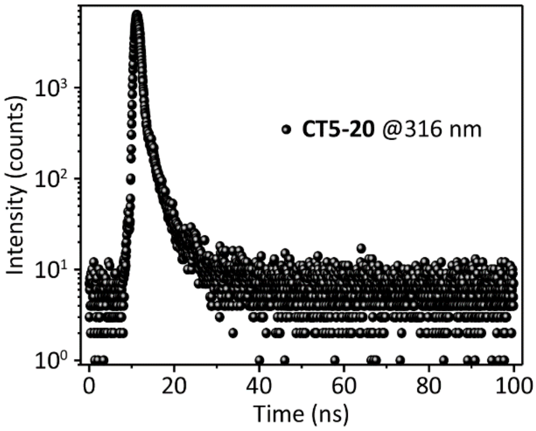

Supplementary Figure 8. Lifetime decay profile of **CT5-20** powder at 316 nm under ambient conditions excited by 248 nm UV light.

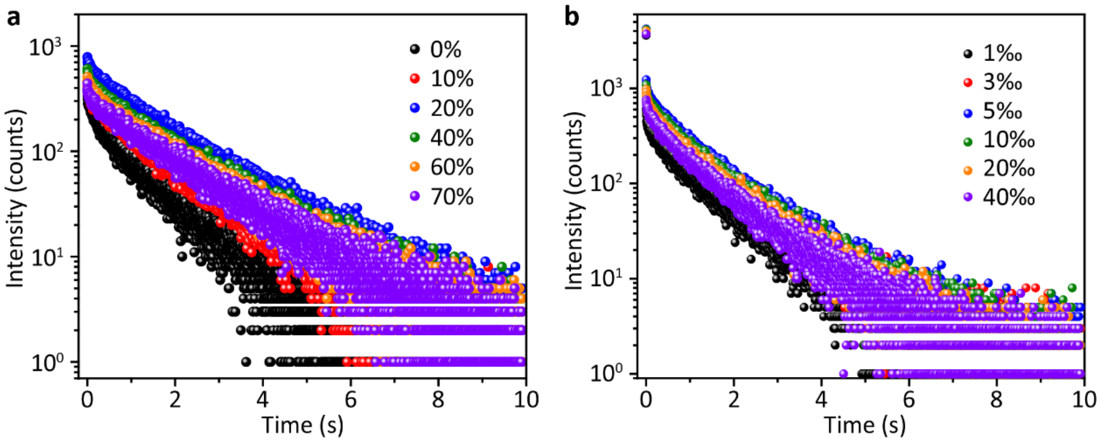

Supplementary Figure 9. Lifetime decay profiles (406 nm) of **CT5-y** powders with various water contents (**a**) and **CTx-0** with different **TMA** contents (**b**) under excitation of 248 nm under ambient conditions.

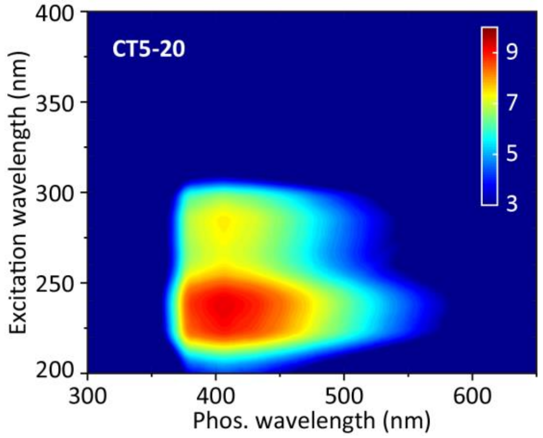

Supplementary Figure 10. Excitation-phosphorescence (phos.) mapping spectrum of **CT5-20** measured under ambient conditions with a delay time of 30 ms.

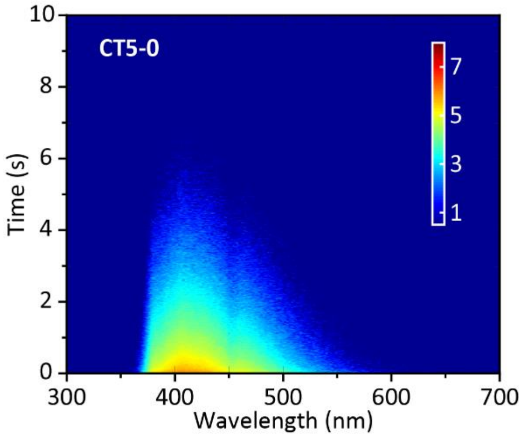

Supplementary Figure 11. Time-resolved emission scanning spectrum of **CT5-0** powder under ambient conditions. The excitation wavelength is 248 nm and the colors changed from blue to red indicate the increase of emission intensity.

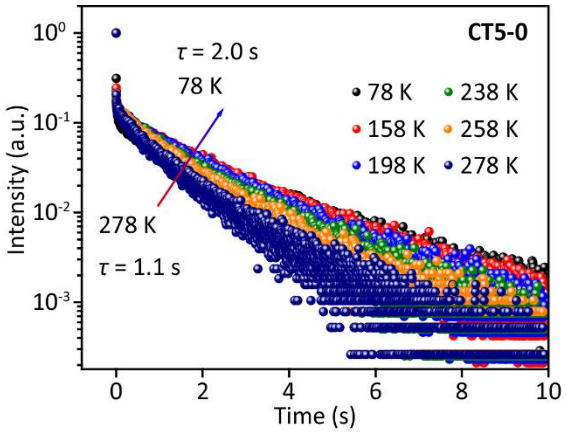

Supplementary Figure 12. Lifetime decay profiles (406 nm) of **CT5-0** powder at different temperature.

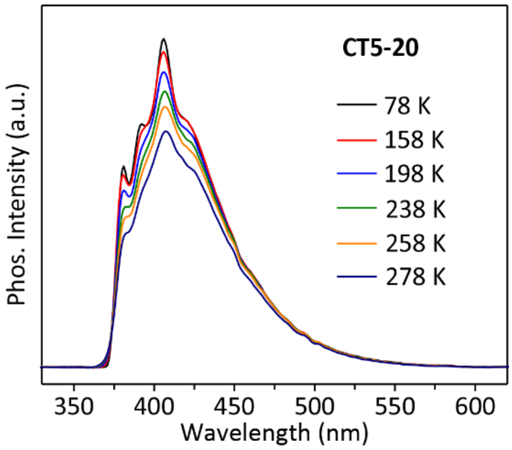

Supplementary Figure 13. Phosphorescent spectra of **CT5-20** powder under excitation of 248 nm with 30 ms delay at temperatures from 78 to 278 K.

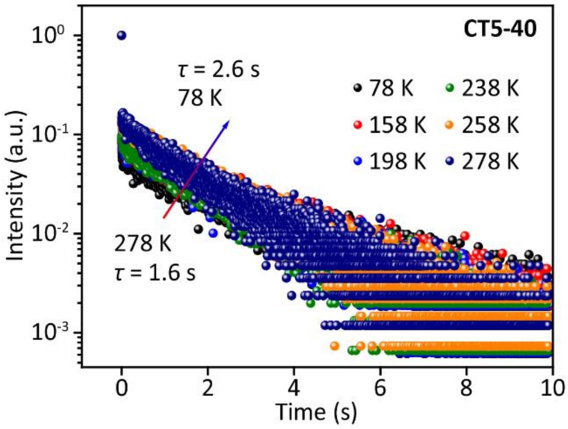

Supplementary Figure 14. Lifetime decay profiles (406 nm) of **CT5-40** powder at different temperature.

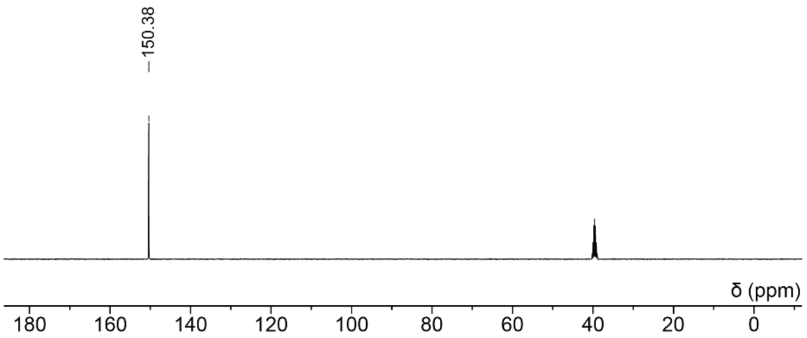

Supplementary Figure 15. ^{13}C-NMR spectrum of **CA** in DMSO-*d*6.

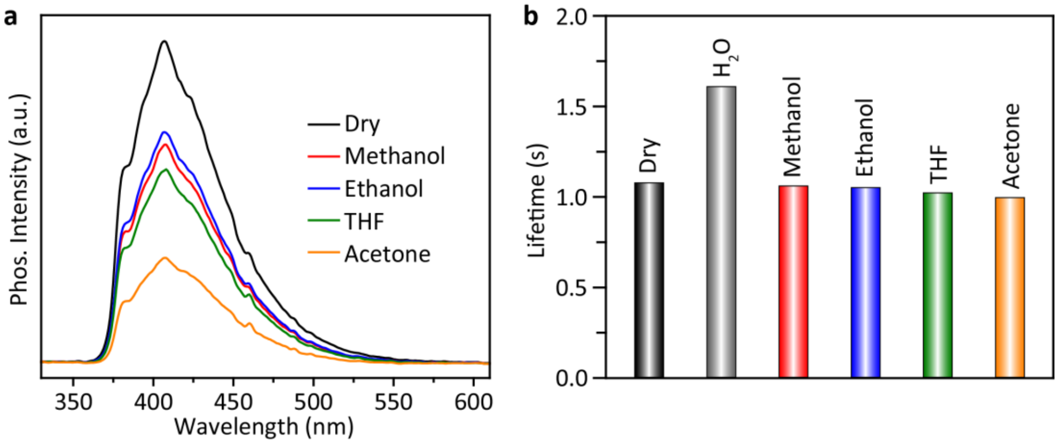

Supplementary Figure 16. Phosphorescent spectra (**a**) and lifetimes (**b**) of the **CT5** powder implemented by various solvents under excitation of 248 nm under ambient conditions.

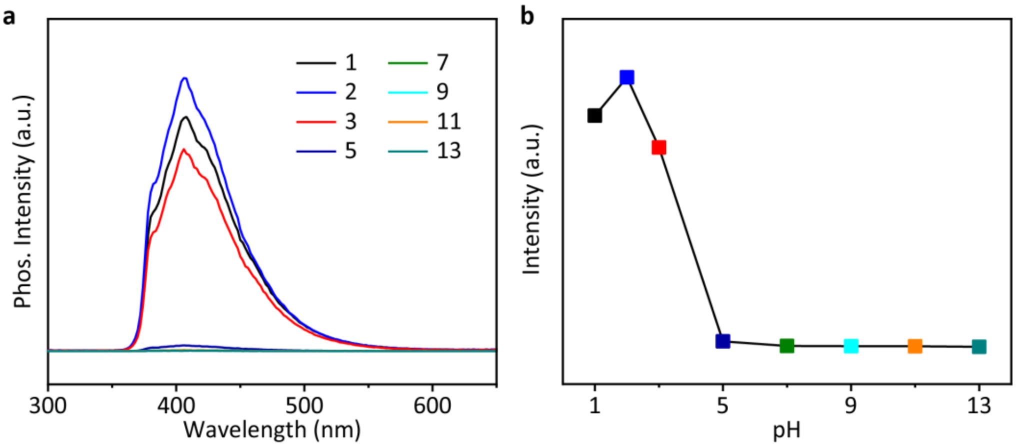

Supplementary Figure 17. Phosphorescent spectra of **CT5-20** prepared by adding **CA** to the aqueous solution of **TMA** with different pH values under ambient conditions. A delay time of 30 ms was applied (**a**). Phosphorescent intensities at 406 nm with different pH values of **TMA** solution (**b**).

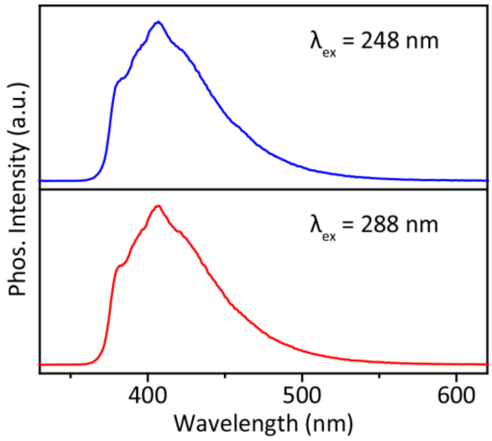

Supplementary Figure 18. Phosphorescent spectra of **CT5-20** excited by 248 and 288 nm under ambient conditions with a delay time of 30 ms.

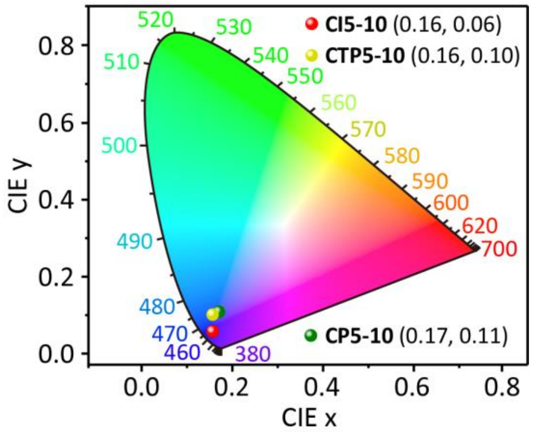

Supplementary Figure 19. CIE coordinates of OURTP emissions of **CI5-10**, **CTP5-10** and **CP5-10**.

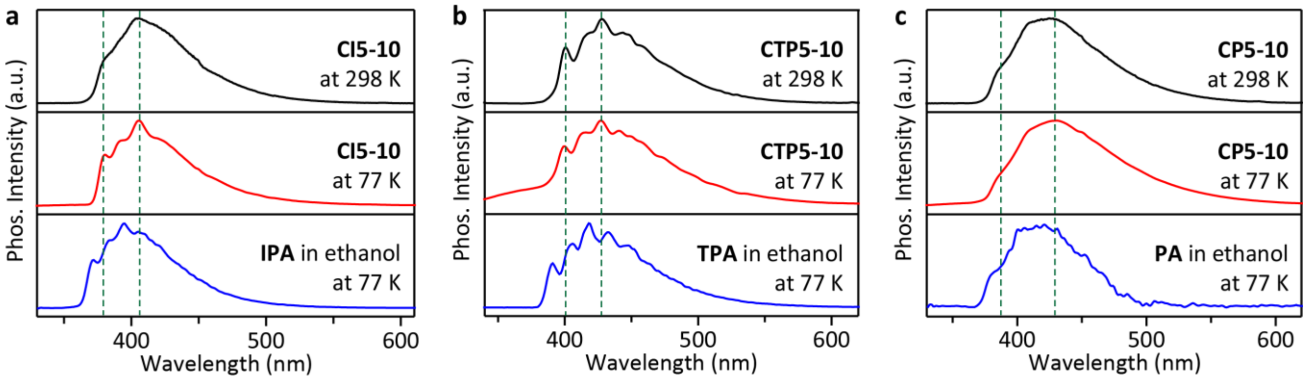

Supplementary Figure 20. Phosphorescent spectra of **CI5-10** at 298 K and 77 K and **IPA** in ethanol at 77K (**a**), **CTP5-10** at 298 K and 77 K and **TPA** in ethanol at 77 K (**b**), and **CP5-10** at 298 K and 77 K and **PA** in ethanol at 77 K (**c**). These spectra are excited by 248 nm with a delay time of 30 ms, except for phosphorscent spectra of **IPA**, **TPA** and **PA** in ethanol which excited by 287, 292 and 285 nm, respectively.

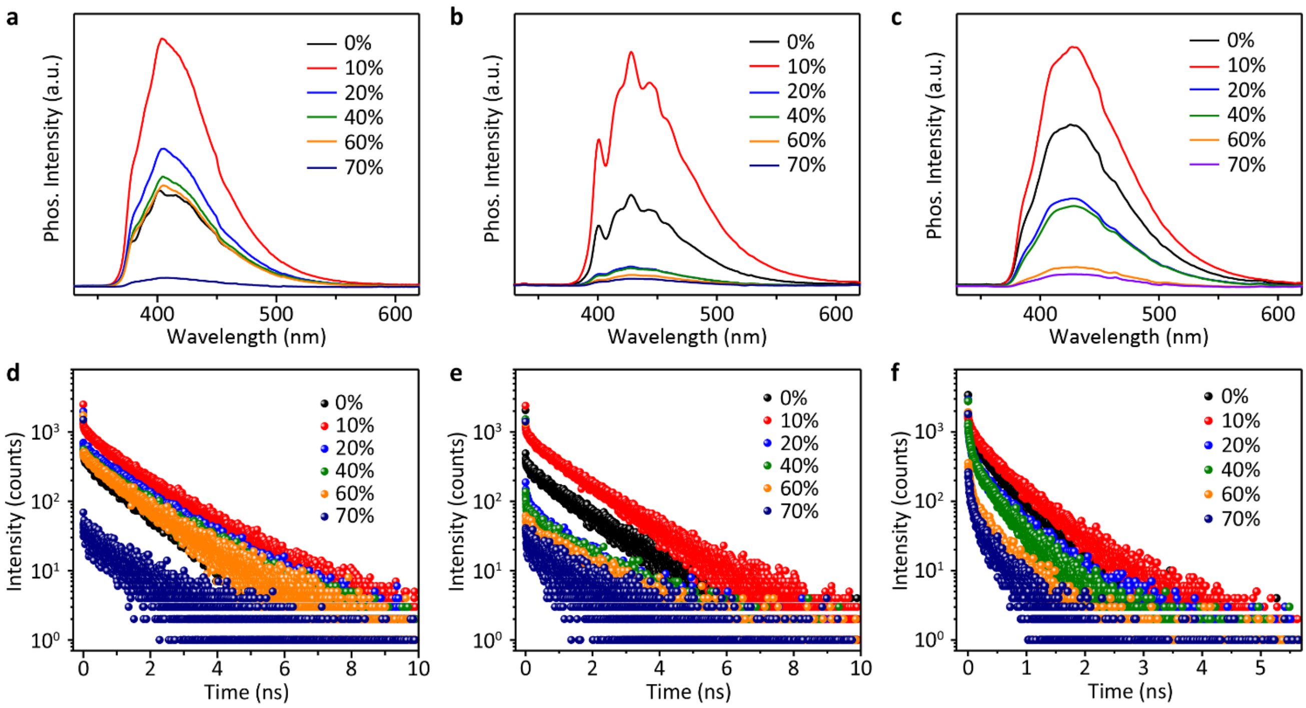

Supplementary Figure 21. Phosphorescent spectra (**a-c**) and lifetime decay profiles (**d-f**) of **CI5**, **CTP5** and **CP5** with different water contents, respectively, under excitation of 248 nm with a delay time of 30 ms under ambient conditions.

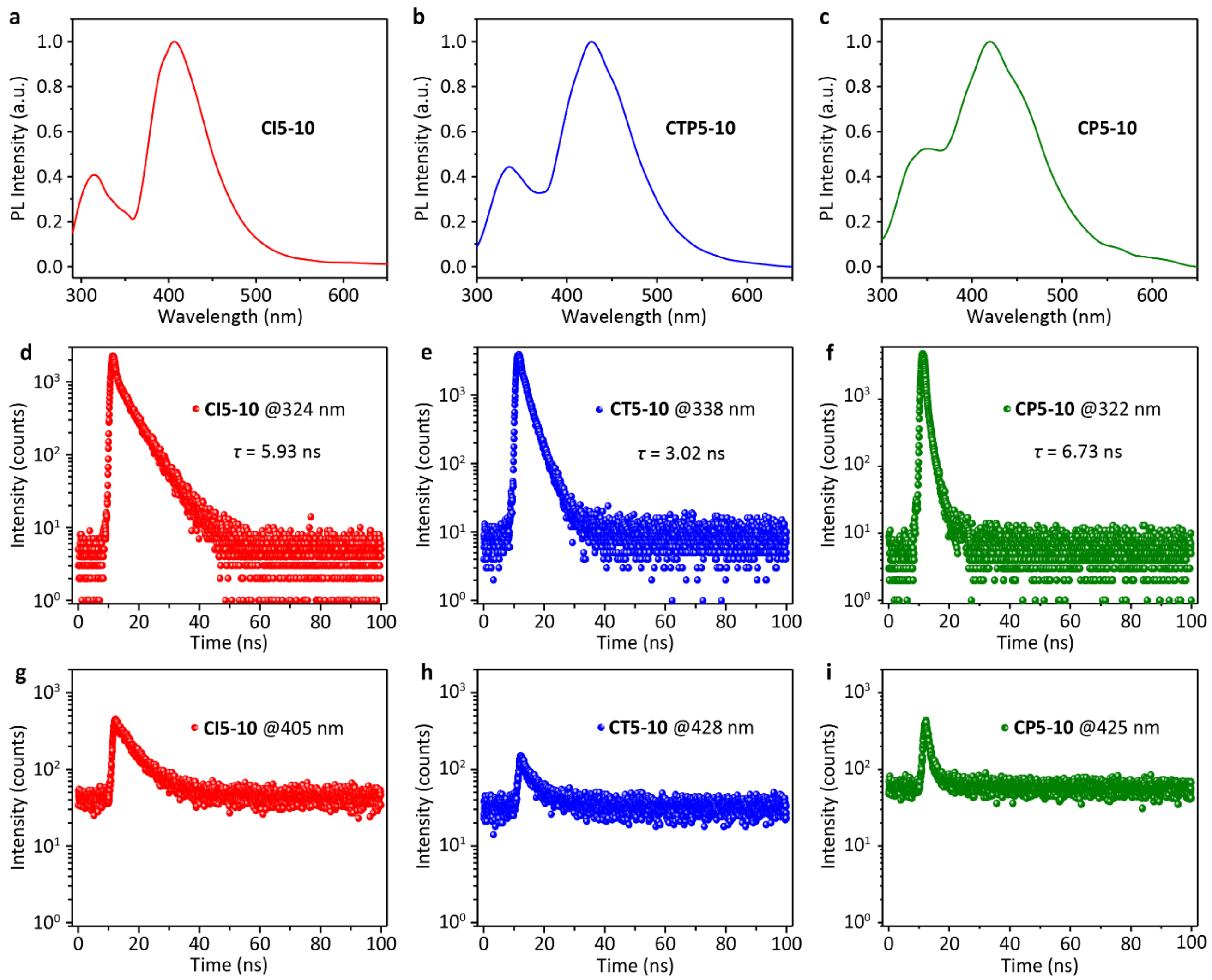

Supplementary Figure 22. Steady-state PL spectra of **CI5-10** (**a**), **CTP5-10** (**b**) and **CP5-10** (**c**). Fluorescent lifetime decay profiles of **CI5-10** at 324 nm (**d**) and 405 nm (**g**), **CTP5-10** at 338 nm (**e**) and 428 nm (**h**), and **CP5-10** at 322 nm (**f**) and 425 nm (**i**) in nanosecond scale. The excitation wavelength is 248 nm.

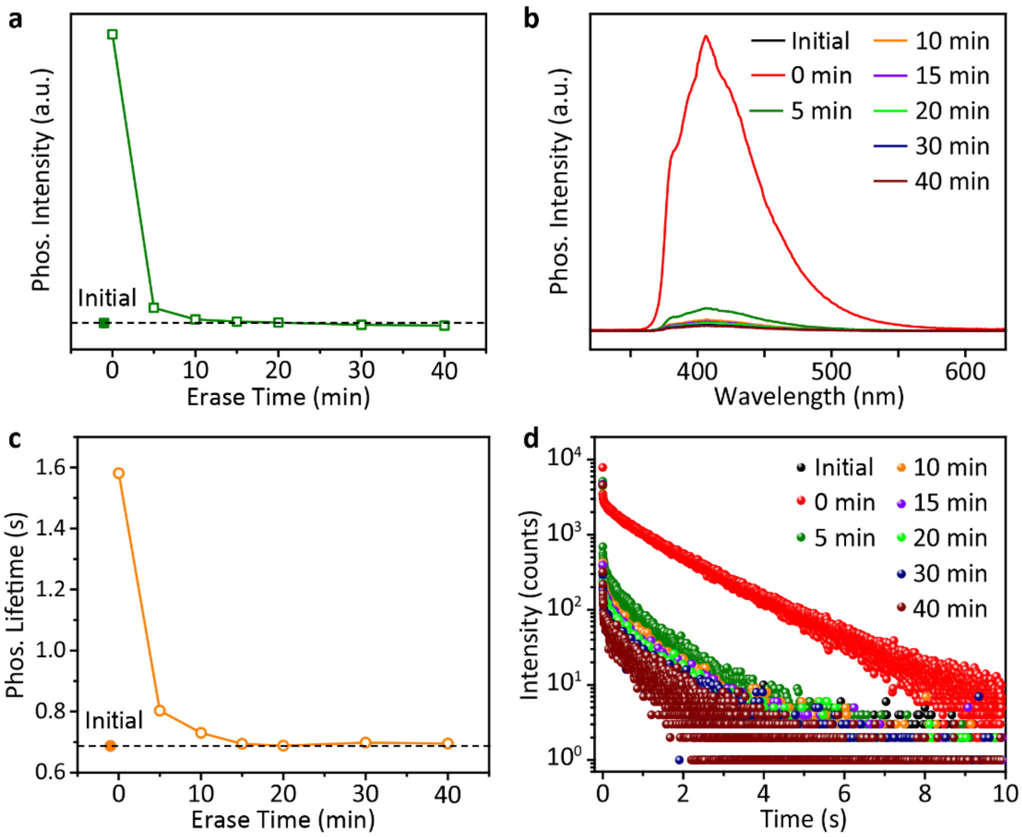

Supplementary Figure 23. Phosphorescent intensity (406 nm) changing (**a**), spectra (**b**), lifetime changing (**c**) and lifetime decay profiles (**d**) of the rewritable encrypted paper at initial state, freshly printed (0 min), and after different erase time using DMSO vapor excited by 248 nm under ambient conditions.

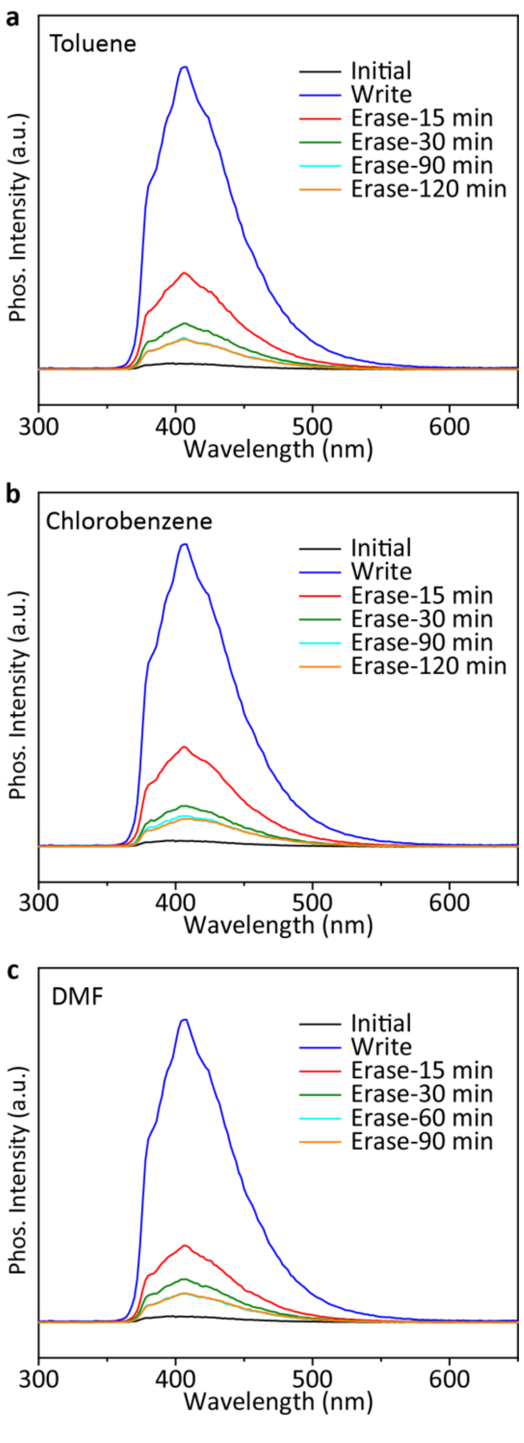

Supplementary Figure 24. Phosphorescent spectra of rewritable paper at initial state, after water writing, and erased by toluene (**a**), chlorobenzene (**b**) and DMF (**c**) vapor fuming for different time. The excitation wavelength is 248 nm and the delay time is 30 ms.

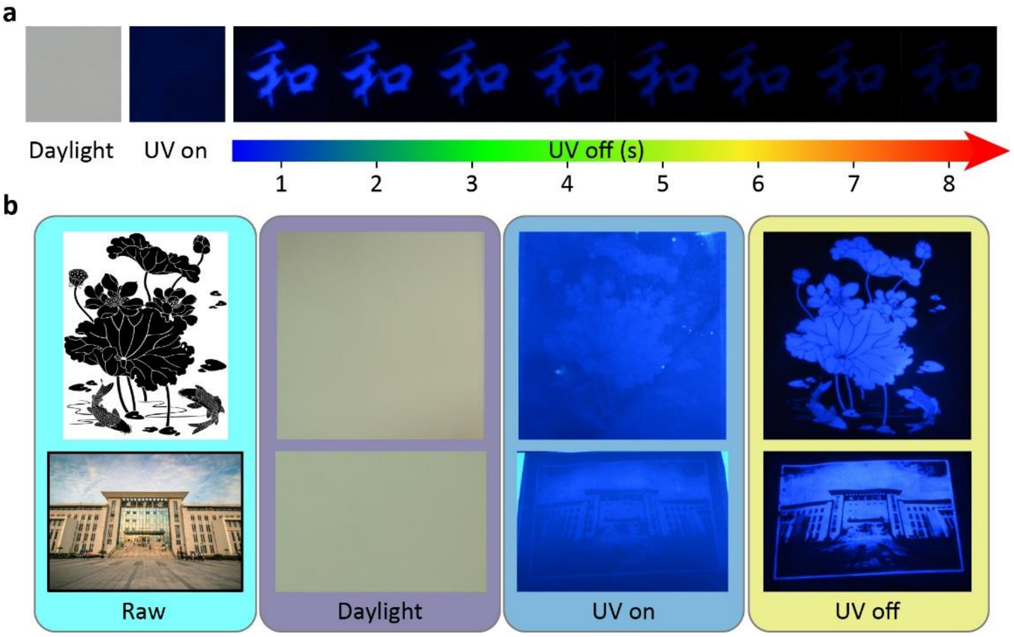

Supplementary Figure 25. Images of Chinese character (**a**) and pictures (**b**) printed on the rewritable lifetime-encrypted paper under daylight, 254 nm UV lamp (UV on) and after removal of the 254 nm UV lamp (UV off).

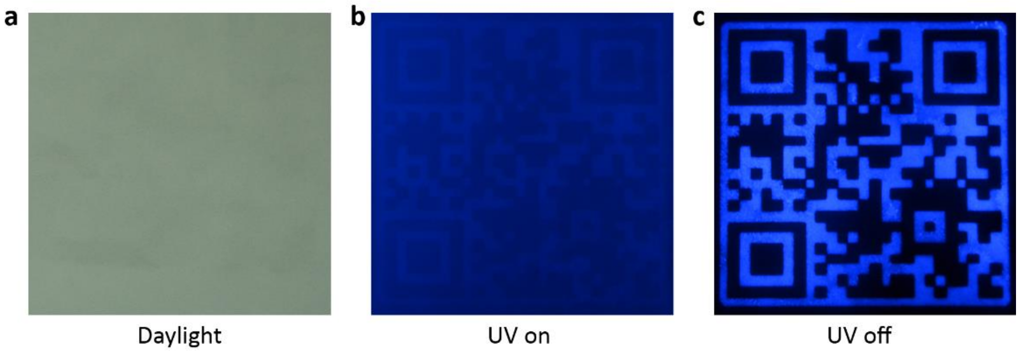

Supplementary Figure 26. Anti-counterfeited two-dimensional code painting. Photographs taken under daylight (**a**), 254 nm UV light (UV on) (**b**) and after removal of the 254 nm UV light (UV off) (**c**).

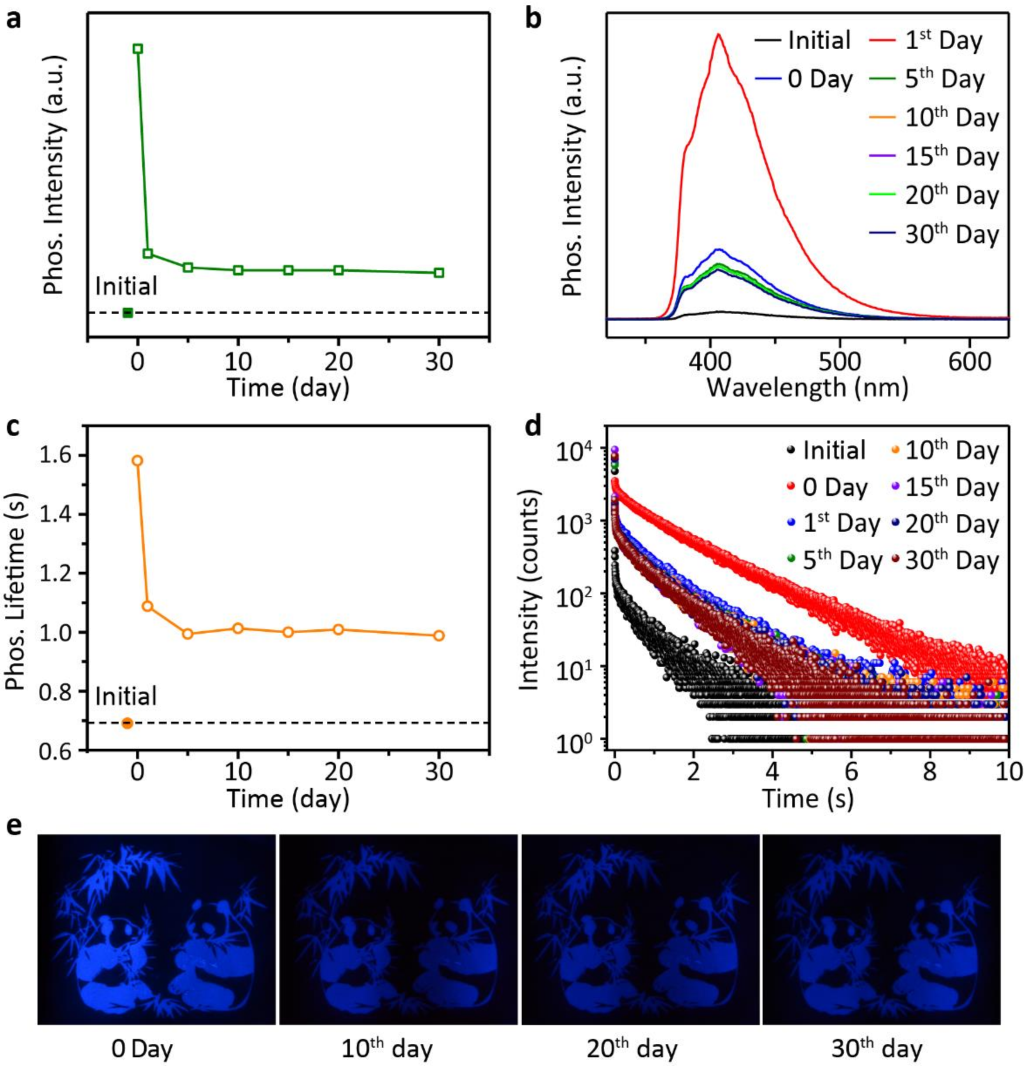

Supplementary Figure 27. Phosphorescent intensity (**a**), spectra (**b**), lifetime (**c**) and decay profiles (**d**) of the rewritable encryption paper at the initial state, freshly printed (0 day), and the 1, 5, 10, 15, 20 and 30 days after printing. The emission wavelength is 406 nm and the excitation wavelength is 248 nm under ambient conditions. **e**, Photographs of the rewritable lifetime-encrypted pattern of freshly printed (0 day) and 10, 20 and 30 days after printing. The photographs were taken with 1 s delay after the removal of 254 nm UV lamp.

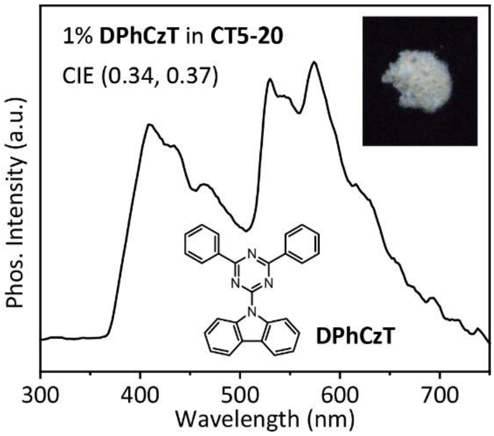

Supplementary Figure 28. Phosphorescent spectrum of 1 wt% **DPhCzT** (4,6-Diphenyl-2-carbazolyl-1,3,5-triazine[1])-doped **CT5-20** collected with a delay time of 30 ms after the excitation of the 254 nm UV light. Inset: photograph of the composite after removing the 254 nm excitation source (up) and the molecular structure of **DPhCzT** (bottom).

3. Supplementary Tables

Supplementary Table 1. Organic ultralong room temperature phosphorescence wavelength (λ_{ph}), lifetimes (τ_{ph}), phosphorescence quantum yields (PhQY) and references (Refs.) of the reported blue organic amorphous afterglow materials.

System		λ_{ph} (nm)	τ_{ph} (s)	PhQY (%)	Refs.
Host	Guest				
β-Estradiol	G1	462	0.66	3.5	2,3
β-Estradiol	G2	468	1.04	6.0	2,3
β-Estradiol	G3	481	1.60	12.1	2,3
β-Estradiol	G4	476	1.39	4.4	2,3
β-Estradiol	G5	490	1.74	7.8	2,3
β-Estradiol	G6	476, 504	3.98	12.9	2,3
β-Estradiol	G7	481	2.72	4.3	2,3
β-Estradiol	G8	492	0.75	2.8	2,3
Choresterol+THEB	G6	480	1.4	3.1	4
PVA	G9	480	0.75	11.2	5
PVA	G10	415	0.34	-	5
PVA	G11	420	0.55	-	5
PVA	G12	435	0.23	-	5
PVA	G13	470	0.51	-	5
PVA	G14	480	0.54	-	5
PVA	G15	450	1.21	-	6
PVA	G16	420	0.24	-	6
$nSiO_2$	m-CDs	461, 518	0.69, 0.70	-	7
Urea, Biuret	NCD1	430, 500	1.11, 0.53	-	8
Urea, Biuret	NCD2	489, 502	1.11, 0.70	-	8
Urea, Biuret	NCDs	490	1.06	7.0	9
CA	CDs	480	0.69	-	10
CA	a-CDs	475	0.78	-	10
CA	c-CDs	430	0.93	-	10
P1		434	0.12	6.40	11
P2		425	0.52	17.9	11
P3		427	0.54	15.4	11
P5		497	0.22	5.4	11
P7		418	0.20	5.2	11
P8		424	0.11	4.0	11
P9		433	0.20	30.6	11
PSS350000		496	1.00	-	12
Carbon dots		455	1.21	3.4	13
**CT5-20**		406	1.67	46.1	This work

Supplementary Table 2. Fluorescent wavelength (λ_{fl}) and lifetimes (τ_{fl}), and phosphorescent wavelength (λ_{ph}), lifetimes (τ_{ph}) and quantum yields (PhQY) of **CA**, **TMA**, **CT5-0** and **CT5-20**.

Material	State	Fluorescence		Phosphorescence		
		λ_{fl} (nm)	τ_{fl} (ns)	λ_{ph} (nm)	τ_{ph} (s)	PhQY (%)
**CA**	Solution[a]	-	-	402	-	-
	Powder	312	5.92	392	0.36	-
**TMA**	Solution[a]	300	-	399	-	-
	Crystal[b]	384	-	524	0.15	2.2
**CT5-0**	Powder	316	4.87	406	1.13	9.3
**CT5-20**	Powder	316	5.32	406	1.67	46.1

[a]Measured at 77 K in ethanol (10 μM). [b]According to literature 14.

Supplementary Table 3. Singlet-triplet splitting energies (E_{S1}-E_{Tn}) and SOC constants from S_1 to T_n of **TMA** and **CA**. The efficient intersystem crossing channels with $|E_{S1}$-$E_{Tn}|$ < 0.37 eV were highlighted in red.

Molecule	Transition	E_{S1}-E_{Tn} (eV)	SOC (cm^{-1})
**TMA**	$S_1 \rightarrow T_1$	0.95	10.67
	$S_1 \rightarrow T_2$	0.41	5.30
	$S_1 \rightarrow T_3$	0.38	2.35
	$S_1 \rightarrow T_4$	0.31	2.20
	$S_1 \rightarrow T_5$	0.24	2.18
	$S_1 \rightarrow T_6$	0.23	4.45
	$S_1 \rightarrow T_7$	-0.02	2.41
	$S_1 \rightarrow T_8$	-0.60	7.79
	$S_1 \rightarrow T_9$	-0.61	9.64
	$S_1 \rightarrow T_{10}$	-0.73	6.28
**CA**	$S_1 \rightarrow T_1$	1.04	26.42
	$S_1 \rightarrow T_2$	0.47	13.61
	$S_1 \rightarrow T_3$	0.47	9.51
	$S_1 \rightarrow T_4$	0.31	15.35
	$S_1 \rightarrow T_5$	0.26	2.98
	$S_1 \rightarrow T_6$	0.26	10.60
	$S_1 \rightarrow T_7$	0.10	0.59
	$S_1 \rightarrow T_8$	0.06	4.06
	$S_1 \rightarrow T_9$	-1.23	1.04
	$S_1 \rightarrow T_{10}$	-1.23	6.48

Supplementary Table 4. Phosphorescent lifetimes (τ_{Ph}) and intensities (I_{Ph}) of **CT5-0**, **CT5-20** and **CT5-40** at different temperatures from 278 to 78 K under excitation of 248 nm after a delay time of 30 ms.

	Material	278 K	258 K	238 K	198 K	158 K	78 K
τ_{Ph} (s)	**CT5-0**	1.13	1.29	1.40	1.59	1.82	2.01
	**CT5-40**	1.60	1.67	1.80	2.02	2.20	2.56
I_{Ph} (x 10^4 counts)	**CT5-0**	0.89	1.06	1.22	1.47	1.67	2.07
	**CT5-40**	0.96	0.95	1.02	1.15	1.23	1.34
	**CT5-20**	0.98	1.08	1.15	1.23	1.31	1.36

Supplementary Table 5. Phosphorescence properties of **CI5** (403 nm), **CTP5** (428 nm) and **CP5** (425 nm) with different water content upon excitation of 248 nm under ambient conditions.

Composites	Water content (wt%)	τ_1 (s)	A_1 (%)	τ_2 (s)	A_2 (%)	τ_{ave} (s)[a]	I_P (a.u.)[b]
**CI5**	0	1.22	100	-	-	1.22	1.14
	10	1.36	100	-	-	1.36	2.96
	20	1.25	100	-	-	1.25	1.65
	40	1.21	100	-	-	1.21	1.32
	60	1.20	100	-	-	1.20	1.21
	70	1.04	100	-	-	1.04	0.12
**CTP5**	0	1.25	100	-	-	1.25	0.93
	10	1.33	100	-	-	1.33	2.37
	20	1.12	100	-	-	1.12	0.21
	40	1.08	100	-	-	1.08	0.19
	60	1.06	100	-	-	1.06	0.12
	70	0.96	100	-	-	0.96	0.09
**CP5**	0	0.10	18.88	0.54	81.12	0.52	0.96
	10	0.14	16.75	0.60	83.25	0.58	1.41
	20	0.08	15.91	0.48	84.09	0.47	0.53
	40	0.06	16.81	0.41	83.19	0.40	0.48
	60	0.05	14.39	0.36	85.61	0.35	0.13
	70	0.04	24.25	0.33	75.75	0.32	0.09

[a]Average lifetime calculated by the function of $\tau_{ave} = (\tau_1^2 A_1 + \tau_2^2 A_2)/(\tau_1 A_1 + \tau_2 A_2)$. [b]Normalized phosphorescent intensity.

Supplementary Table 6. Phosphorescent lifetimes (τ_{Ph}) and intensities (I_{Ph}) of the rewritable encrypted paper at 406 nm at initial state, freshly printed (0 min), and after different erase time upon excitation of 248 nm under ambient conditions.

	Initial	0 min	5 min	10 min	15 min	20 min	30 min	40 min
τ_{Ph} (s)	0.68	1.59	0.80	0.73	0.70	0.69	0.70	0.70
I_{Ph} (×10^4 counts)	0.42	15.92	1.23	0.61	0.49	0.44	0.32	0.28

Supplementary Table 7. Phosphorescent lifetimes (τ_{Ph}) and intensities (I_{Ph}) of the rewritable encrypted paper at 406 nm in eight write/erase cycles upon excitation of 248 nm under ambient conditions.

Cycle		1	2	3	4	5	6	7	8
Erase	τ_{Ph} (s)	0.72	0.71	0.56	0.56	0.68	0.73	0.78	0.74
	I_{Ph} (×10^5 counts)	0.05	0.04	0.06	0.05	0.06	0.05	0.06	0.06
Write	τ_{Ph} (s)	1.66	1.65	1.65	1.62	1.67	1.60	1.64	1.59
	I_{Ph} (×10^5 counts)	1.12	1.04	0.95	0.94	0.92	0.78	0.77	0.77

Supplementary Table 8. Phosphorescent lifetimes (τ_{Ph}) and intensities (I_{Ph}) of the rewritable encrypted paper at 406 nm in initial state, freshly printed (0 day), and the 1st, 5th, 10th, 15th, 20th and 30th day after printing when excited by 248 nm under ambient conditions.

System	Initial	0 Day	1st Day	5th Day	10th Day	15th Day	20th Day	30th Day
τ_{ph} (s)	0.68	1.59	1.09	0.99	1.01	1.00	1.01	1.00
I_{Ph} (×10^4 counts)	0.42	15.92	3.91	3.08	2.92	2.92	2.92	2.77

4. Supplementary Notes

Supplementary Note 1: Summary of reported amorphous blue organic ultralong room temperature phosphorescent materials. As the high-lying triplet excited states are extremely unstable, organic afterglow materials with blue emission are hardly available, not to mention long lifetime and high efficiency. Here, we summarized afterglow properties of amorphous blue organic ultralong room temperature phosphorescent (OURTP) materials reported so far. Among these blue afterglow materials, long lifetime of 3.98 s[2,3] and high phosphorescent efficiency of 30.55%[11] were achieved. However, long lifetime and high efficiency have not been achieved simultaneously until this work.

Supplementary Note 2: ^{13}C-NMR spectra of CA and CT5 composites. CA with 3 equivalent carbon atoms has only a single peak at around 150 ppm in ^{13}C-NMR (Supplementary Figure 15). However, in the ^{13}C-NMR spectrum of **CT5-0,** two peaks at 152.3 and 149.8 ppm can be observed, corresponding to carbon atoms with and without H-bonding in **CA**. After water implement, only a single peak at 152.1 ppm appears, indicating that all carbon atoms are in H-bonds.

Supplementary Note 3: Selection of erasing solvents. Other solvents of toluene, chlorobenzene, and N,N-dimethylformamide (DMF) were also tested as the erasing solvent (Supplementary Figure 24). Compared to the rapid erasing effect of DMSO vapor, these solvent vapors are poor to completely erase the pattern even with much longer time up to 120 min. It is speculated that treatment of DMSO vapor can effectively damage the H-bond network constructed by water molecules by partly dissolving the **TMA** molecules to eliminate the phosphorescence.

Supplementary Note 4: Application for white afterglow. With 1 wt% doped **DPhCzT** (a yellow afterglow molecule) in **CT5-20**, the obtained composite material exhibits both deep-blue OURTP emission of **CT5-20** and yellow afterglow of **DPhCzT**, resulting in white afterglow after the excitation of 254 nm UV light (Supplementary Figure 28).

Supplementary References

1 An, Z. et al. Stabilizing triplet excited states for ultralong organic phosphorescence. *Nat. Mater.* **14**, 685-690 (2015).

2. Hirata, S. Recent advances in materials with room-temperature phosphorescence: photophysics for triplet exciton stabilization. *Adv. Opt. Mater.* **5**, 1700116 (2017).

3. Hirata, S. et al. Efficient persistent room temperature phosphorescence in organic amorphous materials under ambient conditions. *Adv. Funct. Mater.* **23**, 3386-3397 (2013).

4. Hirata, S. et al. Reversible thermal recording media using time-dependent persistent room temperature phosphorescence. *Adv. Opt. Mater.* **1**, 438-442 (2013).

5. Su, Y. et al. Ultralong room temperature phosphorescence from amorphous organic materials toward confidential information encryption and decryption. *Sci. Adv*. **4**, eaas9732 (2018).

6. Wu, H. et al. Achieving amorphous ultralong room temperature phosphorescence by coassembling planar small organic molecules with polyvinyl alcohol. *Adv. Funct. Mater.* **29**, 1807243 (2019).

7. Jiang, K., Wang, Y., Cai, C. & Lin, H. Activating room temperature long afterglow of carbon dots via covalent fixation. *Chem. Mater.* **29**, 4866-4873 (2017).

8. Lin, C. et al. Blue, green, and red full-color ultralong afterglow in nitrogen-doped carbon dots. *Nanoscale* **11**, 6584-6590 (2019).

9. Li, Q. et al. Efficient room-temperature phosphorescence from nitrogen-doped carbon dots in composite matrices. *Chem. Mater.* **28**, 8221-8227 (2016).

10. Li, Q. et al. Induction of long-lived room temperature phosphorescence of carbon dots by water in hydrogen-bonded matrices. *Nat. Commun.* **9**, 734 (2018).

11. Ma, X., Xu, C., Wang, J. & Tian, H. Amorphous pure organic polymers for heavy-atom-free efficient room-temperature phosphorescence emission. *Angew. Chem. Int. Ed.* **57**, 10854-10858 (2018).

12. Ogoshi, T. et al. Ultralong room-temperature phosphorescence from amorphous polymer poly(styrene sulfonic acid) in air in the dry solid state. *Adv. Funct. Mater.* **28**, 1707369 (2018).

13. Long, P. et al. Self-protective room-temperature phosphorescence of fluorine and nitrogen codoped carbon dots. *Adv. Funct. Mater.* **28**, 1800791 (2018).

14. Yang, X. & Yan, D. Strongly enhanced long-lived persistent room temperature phosphorescence based on the formation of metal–organic hybrids. *Adv. Opt. Mater.* **4**, 897-905 (2016).

15. Gu, L. et al. Colour-tunable ultra-long organic phosphorescence of a single-component molecular crystal. *Nat. Photon*. **13**, 406-411 (2019).
